# Construction and Validation of the Research Misconduct Scale for Social Science University Students

**DOI:** 10.3389/fpsyg.2022.859466

**Published:** 2022-05-09

**Authors:** Saba Ghayas, Zaineb Hassan, Sumaira Kayani, Michele Biasutti

**Affiliations:** ^1^Department of Psychology, University of Sargodha, Sargodha, Pakistan; ^2^Department of Psychology, Zhejiang Normal University, Jinhua, China; ^3^Department of Philosophy, Sociology, Education, and Applied Psychology, University of Padova, Padova, Italy

**Keywords:** research misconduct, exploratory factor analysis, validation, confirmatory factor analysis, academic dishonesty, psychometric properties, scale development

## Abstract

The current study aims to construct and validate a measure of research misconduct for social science university students. The research is comprised of three studies; Study I presents the scale construction in three phases. In Phase I, the initial pool of items was generated by reviewing the literature and considering the results of semi-structured interviews. Phase II involved a psychometric cleaning of items, after which 38 items were retained. In Phase III, those 38 items were proposed to 652 university students, and data were exposed to exploratory factor analysis, which extracted a one-factor structure with 15 items and 55.73% variance. Study II confirmed the factorial structure of the scale using an independent sample (*N* = 200) of university students. Confirmatory factor analysis of the scale demonstrates a good model fit to the data with the one-factor structure established through the exploratory factor analysis. The scale exhibits good internal consistency, with a Cronbach’s alpha of 0.95. Study III involves validation of the scale, with evidence for convergent validity collected from a sample of university students (*N* = 200). The results reveal that the research misconduct scale has significant positive correlations with academic stress and procrastination and a significant negative correlation with academic achievement. The obtained convergent validity testifies that the scale can be considered a psychometrically sound instrument to measure research misconduct among social science university students.

## Introduction

Misconduct in research and academic dishonesty are important, persistent issues for universities, as most students have engaged in academic misconduct at some point of their careers (Peled et al., 2019). Almost all (92%) surveyed students reported having cheated at least once or knowing someone who had (Eshet et al., 2021). Unethical research practices and research misconduct can also be found among scholars. A study conducted in Africa showed that about 68.9% of a group of researchers admitted being involved in one of the following forms of research misconduct: plagiarism, falsifying data, intentional protocol violations, selective dropping of data, falsification of biosketches, disagreements about authorship, and pressure from study weight (Okonta and Rossouw, 2013). Another report regarding research misconduct in Nigeria revealed that about 54.6% of researchers acknowledged engaging in at least one practice listed under the criteria of research misconduct (Adeleye and Adebamowo, 2012). There are many indices of research misconduct in other countries, including South Korea, Japan, Taiwan, and China (Bak, 2018; Tsai, 2018); thus, considering the evidence of high misconduct rates, research misconduct needs to be comprehensively examined (Steneck, 2006; Steen, 2011).

A crucial point for developing studies to investigate the effects of research misconduct behavior is having the necessary tools for assessing the phenomenon; to our knowledge, such tools are scarce. The present study seeks to answer the call for tools by constructing and validating a measure of research misconduct among social science students and was conducted with the following objectives:

1. To develop a self-report research misconduct scale for university students.
2. To examine the psychometric properties of the research misconduct scale.

The research is comprised of three studies. Study I presents the scale construction in three phases. Study II illustrates the verification of the factorial structure of the scale. Study III demonstrates the convergent validity of the scale. The background section deals with the definition of misconduct in research, terms that can indicate different types of research misconduct, and the factors connected with misconduct in research.

## Background

Ethical standards and morals play a central role in maintaining research integrity in the scientific community. Abiding norms and ethics promote research based on truthful information and knowledge, discouraging scholars from making errors. Despite detailed and comprehensive guidelines on the standards, ethics, and rules to be followed in research, some scholars still become involved in research misconduct. That behavior is also found among university students who have to conduct research in their final years to earn their degrees.

Research misconduct can be defined as a transgression that occurs when a researcher is involved in fabricating data, falsifying data, or plagiarizing ideas and information in a research project, article, or report. The definition of research misconduct can also be extended to involve wrongdoing related to publication, authorship, and standards of confidentiality (American Psychological Association [APA], 2019). According to Okonta and Rossouw (2013), research misconduct involves various malpractices and actions, such as plagiarizing data, falsification, fabrication, and intentionally violating protocols relevant to research procedures and the enrolment of participants. Other issues involve selectively dropping or skipping outlier cases, conflicts regarding authorship, and pressure from those sponsoring the research study (e.g., an organization or pharmaceutical company) to indulge in research wrongdoing. For the past 20 years in particular, research misconduct and research integrity have been widely discussed and sometimes hotly debated on a variety of platforms. According to the Office of Research Integrity (ORI) in the United States, adhering to commonly accepted rules, standards, principles, norms, and morals is called research integrity (Office of Research Integrity, 2001).

To understand research misconduct, terms like fabrication, falsification, and plagiarism have to be defined, as each is a form of research misconduct. Making up results is called fabrication (Yamamoto and Lennon, 2018), which includes creating and reporting false data or information in a study (Office of Research Integrity, 2019). Fabrication can consist of constructing or adding information, observations, or data that were never actually found during data collection or any other research process. Claims and comments made about incomplete or falsified data sets are also considered a form of fabrication (The Pennsylvania State University, 2018). Fabrication of data is an important research misconduct behavior that can be found in scientific research across disciplines. According to Stretton et al. (2012), almost 52.1% of articles from the medical sciences were found to contain incidences of misconduct, including the fabrication of data.

Falsification can also entail the manipulation of materials, instruments, or processes involved in research or excluding information or results so that representation of the actual research work is compromised (De Vries et al., 2006; Steneck, 2006; Krimsky, 2007; Office of Research Integrity, 2019).

Plagiarism refers to the outright theft or the surreptitious, uncredited use of another person’s ideas, work results, or research. It also includes confidential reviews of other research proposals, reports, synopses, and manuscripts. Another core aspect is that research misconduct is performed intentionally and does not involve genuine differences of opinion or honest errors that can occur in the normal course of research. The World Association of Medical Editors (2019) defines plagiarism as the use of others’ distributed and unpublished thoughts, words, or other licensed innovation without authorization and presenting them as novel. Several motivations could induce authors to resort to plagiarism, including the pressure to publish and having substandard research skills (Jawad, 2013).

Clear and explicit examples of research wrongdoing include plagiarism, falsification, and fabrication. However, many other practices can fall into the category of research misconduct because they deviate from ethical standards in research (Eshet et al., 2021). These include misrepresentation of data in publications, selectively reporting results, characterizing results with low power as unfavorable, improper use of funds, violations of safety protocols, gift authorship, conflicts of interest, and duplicate publications (Federman et al., 2003; Maggio et al., 2019; Haven and van Woudenberg, 2021). Although the aspects that characterize misconduct research are well known, the factors that influence misconduct in research have been less intensively scrutinized.

### Factors of Misconduct in Research

There is a growing interest in research regarding the factors that enable misconduct in research, and we argue that academic stress, procrastination, and academic achievement could all be relevant aspects in the construct validity of the research misconduct scale.

Academic stress can lead a student to commit misconduct in research, according to a qualitative study by Devlin and Gray (2007) on why university students plagiarize. Academic and external pressures were cited as reasons for committing this type of misconduct. In this era of heated academic competition, students feel pressure and complain that the workload placed on them by teachers is difficult to manage and argue that stress due to academic workload could be the cause of research misconduct (Khadem-Rezaiyan and Dadgarmoghaddam, 2017). This happens mainly with university students; because teachers want to make the most of their time and do their best for their students, they often assign maximum tasks, research projects, and assignments. This can make it quite challenging for students to complete all tasks with maximum proficiency and details; sometimes, they are unable to meet all their deadlines. Recruiting participants for research is difficult, and a scarcity of participants can induce students to indulge in unethical—or even illegal—means to complete their assignments. The extreme stress of anticipated failure compels them to conduct fake interviews, complete falsified forms, collect fake data, and finally fabricate and plagiarize data. It is evident that academic stress can play a significant role in influencing academic procrastination among students.

Academic procrastination refers to staying away from academic duties for as long as possible, which can cause students to fail to meet their academic requirements (Ferrari et al., 1995). Several studies have shown that students who demonstrate a careless academic attitude face a variety of negative effects of procrastination (Kandemir, 2010). This kind of educational carelessness inevitably leads to adverse outcomes, such as failing exams (Ferrari et al., 1995; Knaus, 1998), falling behind the rest of the class (Rothblum et al., 1986), and skipping classes and dropping out of school (Knaus, 1998). Ferrari (2004) found that postponing starting or completing an academic task is one of the primary characteristics of academic procrastination, regardless of the student’s intention of ultimately doing the work. Because students who procrastinate begin to work later than those who do not procrastinate, they run out of time to complete work, even something as important as a thesis, before the deadline (Schouwenburg and Groenewoud, 2001). Procrastinating behavior leaves students in a situation where they find themselves out of time and resources. We can assume that students might find it easier to plagiarize, fabricate, or falsify data than do actual work and thus commit research misconduct. Procrastination can have a significant impact on students’ lives, as it can result in low grades that impact various aspects of life and have objectively negative outcomes like poor academic performance (Hussain and Sultan, 2010). In terms of research, procrastination can be classified as a negative attitude. Research needs to be performed through proper planning, design, and investigation rather than being rushed. It demands the investment of adequate time and effort to achieve the best and most accurate outcomes. With continued procrastination, students are left only with the option of faking or plagiarizing by simply fabricating or copying and pasting others’ research results. Plagiarism, which is one of the most common forms of research misconduct, has been highly correlated to procrastination among university students (Siaputra, 2013).

Regarding academic achievement and research misconduct, Finn and Frone (2004) found that students with poor academic performance were likely to commit academic misconduct, including plagiarism, fabrication, and falsification of data. Other research has shown that students with high CGPAs or good academic achievements were less likely to commit plagiarism (Guo, 2011).

### Research Misconduct Assessment

As to assessing research misconduct, only a few scales measuring research misconduct among students or researchers are available, such as the academic dishonesty scale (Bolin, 2004) and a scale examining the perceptions of research coordinators who manage clinical trials regarding different perspectives on misconduct (Broome et al., 2005).

The academic dishonesty scale (Bolin, 2004) is composed of nine items describing behaviors like “copied material and turned it in as your own work,” “used unfair methods to learn what was on a test before it was given,” “copied a few sentences of material from a published source without giving the author credit,” and “cheated on a test in any way.” The academic dishonesty scale is not focused solely on research but also considers other behaviors that could involve students’ academic tasks.

The different perspectives of the misconduct questionnaire (Broome et al., 2005) were used as part of a broader study aimed at analyzing scientific misconduct from a research supervisor’s or coordinator’s point of view. However, it does not measure the tendency for deliberately committing or slowly becoming involved in research misconduct. Previously, information was collected by directly asking scientists if they were involved in any kind of research misconduct (i.e., fabrication, falsification, and plagiarism) in surveys or interviews. Questions regarding primary forms of misconduct were asked, ignoring minor details like authorship credit details, data screening, and violation of protocols (Greenberg and Goldberg, 1994; Martinson et al., 2005). To the best of our knowledge, there are currently no instruments for measuring the level of research misconduct, and the present study intends to fill this gap by developing a tool for that purpose.

## Materials and Methods

This study aims to construct and validate a measure of research misconduct for students, including both quantitative and qualitative research misconduct, although they are different in nature. This paper reports on three studies.

(1) Study I focuses on the construction of a scale in three phases (generation of item pool, item cleaning, and exploration of factor structure). In Phase I, the initial pool of items was generated by reviewing the literature and considering the results of semi-structured interviews. Phase II involved psychometric cleaning of items, after which 38 items were retained. In Phase III, the items were proposed to 652 university students, and an exploratory factor analysis (EFA) was calculated.
(2) In Study II, the factorial structure was tested through a confirmatory factor analysis (CFA) on data collected from an independent sample of 200 university students.
(3) Study III includes validation (convergent validity) of the scale through the administration of questionnaires to a sample of 200 university students. The correlation between research misconduct and the following tools were used to provide evidence for convergent validity: academic procrastination scale (short form), academic stress scale, and academic achievements as measured by cumulative grade point average (CGPA).

The institutional review board of the University of Sargodha gave ethical approval for the research. After that approval was granted, a letter was submitted to the head of the Department of Psychology asking for permission to gather data from students. Once that permission was obtained, questionnaires were presented to the participants, who were assured that the data collected would be used solely for research purposes and that their identities and personal information would be kept confidential. Participants were approached personally and given detailed instructions regarding the purpose of the study and how to complete the questionnaires. Informed consent was obtained from the participants before data collection. Participants were thanked for their support in the research and were provided contact details if they wanted to obtain any further information about the research. Data collection began in September 2021 and finished in November 2021.

### Study I: Development of Research Misconduct Scale

Study I consists of the construction of the scale and has three phases. The first phase identified the pool of items for the research misconduct scale, the second involved a psychometric cleaning of items, and the third involved EFA and psychometric properties.

#### Phase I: Initial Item Pool for Research Misconduct Scale

An initial pool of items was generated using empirical and deductive approaches. With the literature review in mind, items for the research misconduct scale were generated in English. All available literature related to research misconduct was reviewed, giving access to a wide array of concepts and ideas of research misconduct. Qualitative, unstructured individual interviews were also carried out by the researchers to expand their knowledge and obtain subjective viewpoints about research misconduct. Item pool generation was completed by using the following steps:

1. Literature-based: different domains of research misconduct were analyzed to identify aspects that could be used in a single psychometric measure of research misconduct and might provide a quantitative score of research misconduct as a whole.
2. To obtain an understanding of research misconduct and generate additional items for the scale, detailed unstructured interviews were carried out with professors (*n* = 20) and students (*n* = 50) from the University of Sargodha in Punjab. Interview participants were assured of the confidentiality of any information they provided. After they provided signed informed consent, they were asked to report the type of student misconduct incidents they regularly face, different examples of research misconduct, and unique cases of research misconduct. Students enrolled in MPhil and BS (Hons) programs were interviewed regarding the types of misconduct they had committed or observed in their peers. In addition, students were asked to report any factors that they thought might compel them to indulge in such acts.

#### Phase II: Psychometric Cleaning of Items

1. The initial item pool produced from the literature review and interviews yielded 40 items. After the initial item pool was generated, experts (*n* = 10) in psychology and psychometry offered reviews and opinions of the suitability of each item for the research misconduct scale. These expert opinions ensured the relevance and applicability of items to the target population. Information about the purpose of the scale was provided to the experts, who individually analyzed whether the items were culturally and contextually relevant and suggested any additions to or elimination of items in the scale. In response to the experts’ views, 38 items were retained as best fitting the literature, cultural context, and target population.
2. That final scale of 38 items used a five-point Likert-type approach (1 = strongly disagree to 5 = strongly agree). The five-point response format was chosen based on its property of maintaining a balance between both poles while providing respondents with more freedom to choose from the response range that best depicted their views (Gregory, 2004).

#### Phase III: Exploratory Factor Analysis and Psychometric Properties

##### Participants

A sample of 652 university students (300 male, 352 female) belonging to social sciences departments was recruited for the study through a convenience sampling technique. Only full-time students with at least one research experience were included. Participant age ranged from 20 to 24 (*M* = 21.5, *SD* = 5.12).

##### Results

To determine the final structure of the scale, an EFA was carried out on the sample of 652 participants through principal axis factoring and the direct oblimin method. This rotation method was used based on the assumption that if more factors were yielded, they would share some sort of covariance (Field, 2013). A single factor was clearly obtained, accounting for a substantial amount of variance (55.73%) as you can see in Table 1.

**TABLE 1:** Factor loadings through principal axis factoring for the research misconduct scale (*N* = 652).

	Standardized factor loadings
Item no.	F1
1	0.71
2	0.73
3	0.72
4	0.75
5	0.71
6	0.72
7	0.81
8	0.73
9	0.73
10	0.78
11	0.72
12	0.76
13	0.70
14	0.72
15	0.78
Eigenvalue: 9.91	
% of variance: 55.73	

The Kaiser-Meier-Olkin (KMO) test and Bartlett’s Test of Sphericity were applied to assess whether the sample was adequate. The KMO value was 0.92, showing perfect sample sufficiency and adequacy (Kaiser, 1974). Bartlett’s Test of Sphericity was also significant, indicating that the items are significantly correlated and that the sample is appropriate for further analysis (Field, 2013). According to Coakes and Steed (2003), factor analysis is quite sensitive to assumptions of normality. Therefore, skewness and kurtosis were calculated to assess the normality of the data; good normality was obtained. All the commonalities were considerably high, suggesting that factor analysis could proceed; thus, all variables were selected for further analysis.

The EFA yielded a one-factor solution with a direct oblimin rotation method and eigenvalues > 1.0. The obtained one-factor structure was well defined and interpretable with theoretical reliability and construct relevance. Of 38 items, 15 were retained for their substantial loadings (≥0.70) on a single factor. A single-factor structure was interpreted as satisfactory factor loading and theoretical relevance of all items to the factor.

The following 15 items showed exclusive loading on a single factor: “faking,” “cheating,” “misconducting,” “manipulating,” “plagiarism,” “fabricating,” and “favored authorship” were the hallmarks of the obtained factor. The scale’s Cronbach’s alpha was computed; the value of 0.95 indicated very good reliability and excellent internal consistency.

### Study II: Confirmatory Factor Analysis

#### Participants

A sample of 200 university students (101 male, 99 female) was recruited through a convenience sampling technique, using the same affiliation and criteria as applied in Study I.

#### Results

Based on the initial criteria (i.e., item loading > 0.70), the model obtained through EFA was analyzed *via* CFA; the factor structure obtained showed an excellent fit with the data. The goodness of fit (GFI) value and the comparative fit index (CFI) are fairly close to one, and the value of root mean square error of approximation (RMSEA) is significantly close to zero, indicating a good model fit. The value of chi-square/df is 2.91; as that is less than three, it is considered good (Hatcher and Stepanski, 1994). The final model obtained through CFA consisted of 15 items and presented a good model fit.

**FIGURE 1:**
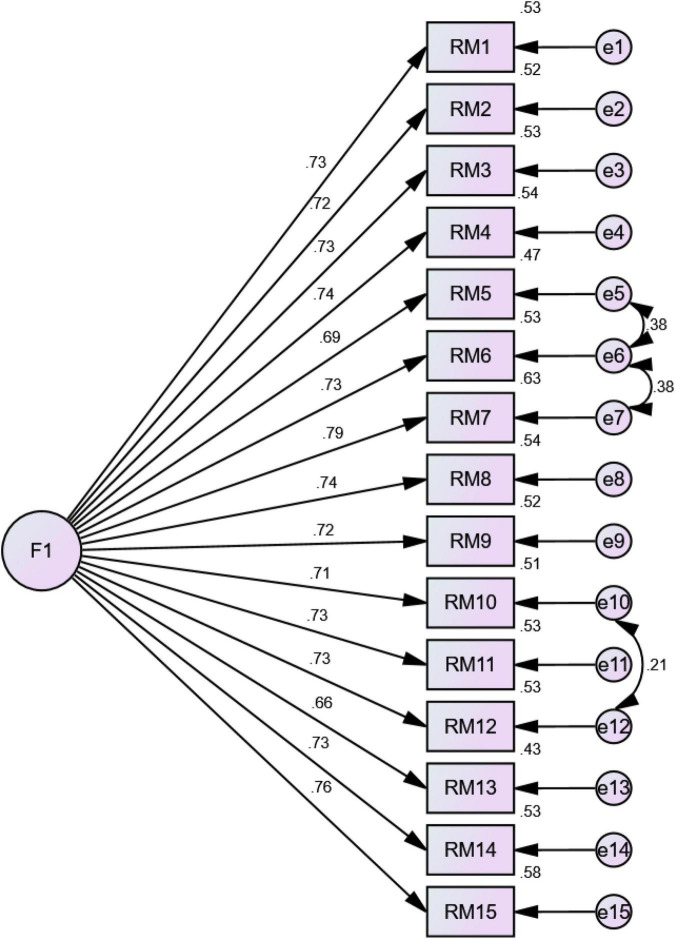
Standardized factor loadings in the CFA of the research misconduct scale.

Table 2 reports the final model obtained through CFA; factor loadings ranged from 0.66 to 0.79. Figure 1 shows the standardized factor loadings in the CFA.

**TABLE 2:** Model fit indices of CFA for research misconduct scale for university students (*N* = 200).

Indexes	Chi-square	*df*	Chi-square/df	CFI	RMSEA	GFI	TLI
Model	245.26	84	2.91	0.92	0.05	0.90	0.91

### Study III: Convergent and Discriminant Validation of Research Misconduct Scale for University Students

Study III was designed to verify the validity for the research misconduct scale for university students. To provide evidence for this validity, the study tested the following hypotheses:

• H1: A positive relationship between research misconduct and academic procrastination would provide evidence of convergent validity.
• H2: A positive relationship between research misconduct and academic stress would provide evidence of convergent validity.
• H3: A negative relationship between research misconduct and CGPA would provide evidence of convergent validity.

#### Participants

A sample of 200 university students (100 male, 100 female) was recruited through a convenience sampling technique, with the same affiliation and criteria as applied in Study I.

#### Instruments

##### Research Misconduct Scale

The research misconduct scale is a 15-item self-report measure (see Appendix 1) developed to examine research misconduct in university students. The response format of the scale is a five-point Likert-type format (1 = strongly disagree, 2 = disagree, 3 = neutral, 4 = agree, and 5 = strongly agree). A high score on the scale represents a high level of research misconduct, while low scores represent low levels of research misconduct. There are no reverse scored items on the scale, which is comprised of only one factor. The Cronbach’s alpha of the reliability index of the scale is 0.95.

##### Academic Procrastination Scale (Short Form)

A short form of the academic procrastination scale (Yockey, 2016), which consists of five items, was used in the present study. The response format of the scale is a five-point Likert-type format (1 = disagree, 5 = agree). There are no reverse scored items on the scale. A high score on this scale represents a high level of procrastination, while low scores represent low levels of procrastination. The scale shows good internal consistency, with a Cronbach’s alpha of 0.87.

##### Academic Stress Scale

Developed by Lin and Chen (2009), the academic stress scale consists of 34 items with responses using a five-point Likert-type format (1 = strongly disagree, 2 = disagree, 3 = do not know, 4 = agree, 5 = strongly agree). High scores on this scale represent high levels of academic stress, and low scores represent low levels of academic stress. There are no reverse scored items on this scale; its Cronbach’s alpha is 0.90, showing good internal consistency.

##### Academic Achievement as Measured by Cumulative Grade Point Average

Student CGPAs were taken as evidence for the convergent validity of the study; CGPAs range between 0.00 and 4.00. Students were asked to provide information about their CGPAs in the previous semester.

#### Results

Table 3 shows correlations between the research misconduct scale, academic stress scale, academic procrastination scale, and CGPA. The research misconduct scale has a significant positive correlation with the academic stress scale (*r* = 0.74, *p* < 0.01) and the academic procrastination scale (*r* = 0.58, *p* < 0.01) but a significant negative correlation with CGPA (*r* = −0.38, *p* < 0.01). The academic stress scale has a significant positive relation with the academic procrastination scale (*r* = 0.67, *p* < 0.01) and a significant negative correlation with CGPA (*r* = −0.25, *p* < 0.05). Finally, the academic procrastination scale has a significant negative correlation with CGPA (*r* = −0.22, *p* < 0.05).

**TABLE 3:** Correlation of research misconduct scale with academic stress scale, academic procrastination scale, and CGPA (*N* = 200).

	RMS	ASS	APS	CGPA
RMS	–	0.74**	0.58**	−0.38**
ASS		–	0.67**	−0.25*
APS			–	−0.22*
CGPA				–

*RMS, Research misconduct scale; ASS, Academic stress scale; APS, Academic procrastination scale; CGPA, Cumulative grade point average.*

**p < 0.01; *p < 0.05.

## Discussion

The present study has illustrated the steps for developing a 15-item self-report measure for research misconduct among students. The items on the scale are general and related to various processes and ethical issues involved in conducting research. The EFA highlighted that the scale is unidimensional and that one factor explained 55.73% of the total variance, while CFA confirmed the one-factor structure obtained through EFA and showed that the model fits for the data and alpha reliability were 0.95, indicating excellent internal consistency (Biasutti and Frate, 2017, 2018).

The research misconduct scale has been validated by testing correlations with the academic stress scale, the academic procrastination scale, and CGPA. Research misconduct had a significant positive correlation with academic stress, and academic procrastination, and a negative correlation with CGPA.

Regarding hypothesis one (“A positive relationship between research misconduct and academic procrastination would provide evidence of convergent validity”), it was assumed that a positive correlation of academic procrastination with research misconduct would provide evidence of convergent validity. The present study’s findings support hypothesis one because research misconduct had a significant positive correlation with academic procrastination. Most of the time, students procrastinate on their academic tasks while prioritizing non-academic activities. Procrastinating behaviors place students in situations where they find themselves out of time and resources; for some, the only solution appears to be inappropriate behaviors like faking and plagiarizing. Plagiarism is one of the most common forms of research misconduct, with several studies (e.g., Siaputra, 2013) finding a high correlation between research misconduct and procrastination in university students. The findings of the present study are in line with another study that suggested a significant positive correlation between plagiarism and academic procrastination (Roig and DeTommaso, 1995), as well as with a panel study of German university students, which revealed that higher levels of academic procrastination results were connected to higher levels of plagiarism, falsification, and data fabrication (Patrzek et al., 2014).

Concerning hypothesis two (“A positive relationship between research misconduct and academic stress would provide evidence of convergent validity”), the fact that research misconduct had a significant positive correlation with academic stress is unsurprising in today’s academic environment (Devlin and Gray, 2007). Keeping in mind the role of academic stress in research misconduct, it was argued that a positive correlation of academic stress with research misconduct would provide evidence of convergent validity. The analysis supports this second hypothesis of the study, which is in line with previous research in which university students noted that academic stress and pressure might be reasons for research misconduct (Khadem-Rezaiyan and Dadgarmoghaddam, 2017). In addition, the findings here are supported by a qualitative study on why university students plagiarize that found academic and external pressures to be reasons behind that type of misconduct (Devlin and Gray, 2007). Students engage in research misconduct when they encounter a task that is more demanding than their capabilities and skills, which places them under stress. Plagiarism is correlated with the difficulty and nature of students’ tasks, which can also be considered connected to academic stress (Tindall and Curtis, 2020).

With regard to hypothesis three (“A negative relationship between research misconduct and CGPA would provide evidence of convergent validity”), it was conjectured that a negative correlation of CGPA with research misconduct would provide evidence of convergent validity. The study’s findings supported the third hypothesis and revealed that research misconduct has a significant negative correlation with CGPA. Conducting research demands a mix of convergent and divergent abilities such as critical thinking, analyzing and comparing situations, planning, scheduling, comprehension, and creativity. Not everyone can think outside the box, which is crucial for designing research. These skills are also related to academic scores. High IQ is a determinant of high scores on certain kinds of tests, but university courses now focus on skills that go beyond merely cramming for exams. Knowing the practical applications of knowledge is more demanding than simply digesting an entire syllabus. Hence, the focus of exams is now more on applied principles of knowledge. Students who are not able to apply knowledge in practical terms do not score well. Low scorers also fail to conduct good research, as they do not have the prerequisite knowledge and skills for conducting good research. The findings of the present study are aligned with those reported by Comas-Forgas and Sureda-Negre (2010), who stated that students with low academic success are more likely to plagiarize. As plagiarism is one of the significant components of research misconduct, this evidence can be taken to support the hypothesis. Similarly, research has shown that students with higher CGPAs or good academic performance were less likely to commit plagiarism (Guo, 2011).

### Limitations

The present study has certain limitations as to participants. The study sample was a convenience sample and consisted solely of students from one university in Punjab. The sample was not representative of the total student population, so the results cannot be generalized to all students. The students available for this study did not represent the actual percentages of students from different religions and races. In addition, only students aged 20 to 24 were included.

The techniques for scale refinement—EFA and CFA—that were used in the present study were specific to sample size, and it is advisable to confirm or refine the findings in further research using a larger sample. All the constructs of the present study were measured through self-report measures, which might have resulted in inflated correlation among the study’s variables. However, an inspection of the correlation matrix among the variables of the present study revealed that none of the correlations was too high, which reduces the likelihood of common method bias.

### Educational Implications

Several educational implications of this study could be discussed. Regarding the positive correlation between research misconduct and academic stress, it could be suggested to university professors do not put students too much under pressure because this could generate wrongdoing behaviors. Prevent to generate stress could be a strategy to avoid misconduct behaviors in students. Other actions could be taken to discourage academic procrastination, which is in correlation with research misconduct. The variables of academic procrastination could be examined to identify factors to be controlled in the educational process of university students.

### Future Research

Future studies should explore other potential correlations of research misconduct to expand its nomological network. Research misconduct could be studied in association with aspects such as personality traits and academic ethics. The research design used here was cross-sectional and does not provide any causal evidence. Therefore, investigating research misconduct using an experimental research design is suggested. Future research should verify the test-retest reliability of the research misconduct scale. For this purpose, a longitudinal research design should be adopted to assess the temporal stability of research misconduct as operationalized through the research misconduct scale. Future studies should investigate the criterion-related validity of the research misconduct scale by examining the concurrent validity in cross-sectional designs and the predictive validity in longitudinal designs. In addition, different populations of students could be involved, including doctoral students.

### Applications of the Research Misconduct Scale

The research misconduct scale developed and validated in the present study opens new avenues of research. Often, research misconduct stays in the dark and is not reported to save an institution’s integrity. This scale should be used in future studies to strengthen research integrity and the scientific community’s ability to assess research misconduct and its various correlations. Furthermore, cross-cultural research on misconduct might help students evaluate the actual and perceived seriousness and consequences of research misconduct.

The research misconduct scale could be used in a triangulation approach for assessing the causes and consequences of research misconduct among various institutes. Both instructors’ and students’ perceptions of research misconduct could be analyzed. More specifically, the administration of the research misconduct scale in various universities might help supervisors, policymakers, curriculum developers, and administrators identify specific factors related to research misconduct and take appropriate actions to stop or at least reduce the practice.

## Data Availability Statement

The raw data supporting the conclusions of this article will be made available by the authors, without undue reservation.

## Ethics Statement

Ethical review and approval were not required for the study on human participants in accordance with the local legislation and institutional requirements. The patients/participants provided their written informed consent to participate in this study.

## Author Contributions

SG: conceptualization, writing original draft, and supervision. SK: methodology and formal analysis. ZH: formal analysis. MB: critical rewriting and supervision. All authors have read and agreed to the published version of the manuscript.

## Conflict of Interest

The authors declare that the research was conducted in the absence of any commercial or financial relationships that could be construed as a potential conflict of interest.

## Publisher’s Note

All claims expressed in this article are solely those of the authors and do not necessarily represent those of their affiliated organizations, or those of the publisher, the editors and the reviewers. Any product that may be evaluated in this article, or claim that may be made by its manufacturer, is not guaranteed or endorsed by the publisher.

## Appendix

**Appendix 1 |** The research misconduct scale for students.

Please indicate the extent of your agreement or disagreement with the statements by using the following scale:

**Table:**

	1	2	3	4	5
	Strongly disagree	Disagree	Neutral	Agree	Strongly agree
(1) Rather than putting in effort, I would prefer to pay someone to get my research projects or some of their parts done	1	2	3	4	5
(2) I have reported fake research studies in my research project	1	2	3	4	5
(3) To please someone, I might add their name as an author without a significant contribution to my research	1	2	3	4	5
(4) If collecting data from a sample is difficult (challenging population, poor response rate, etc.), I might complete data collection tools (questionnaires, interviews, checklists, etc.) myself	1	2	3	4	5
(5) If an author is taking too much time to respond, I might be compelled to use the research tool without his or her permission	1	2	3	4	5
(6) Performing the main analysis without checking its assumptions is not misconduct	1	2	3	4	5
(7) If the results do not turn out as expected, I might manipulate data to send it in my desired direction	1	2	3	4	5
(8) I am not particularly concerned about keeping research information (demographics, responses, etc.) confidential	1	2	3	4	5
(9) If there was no risk of being caught, I would not mind claiming someone else’s work as my own	1	2	3	4	5
(10) It is fine to report high reliability even if it is actually lower than is required in my research	1	2	3	4	5
(11) I have reported non-significant findings as significant ones	1	2	3	4	5
(12) I have manipulated demographics to balance the ratio between groups in my research	1	2	3	4	5
(13) In my opinion, adding fake references in research is not misconduct	1	2	3	4	5
(14) If there is no fear of being caught, I might easily report false results in my research	1	2	3	4	5
(15) I have mixed original and fake data (questionnaires, interviews, documented records, etc.) during data collection in my research	1	2	3	4	5
